# Potent Rifampicin derivatives can clear MRSA infections at single low doses when concomitantly dosed with Vancomycin

**DOI:** 10.1038/s41429-023-00663-6

**Published:** 2023-10-23

**Authors:** Thomas Nittoli, Anna Brotcke Zumsteg, Abira Bandyopadhyay, Stephanie Federici, Alida Coppi, Susan Jorgenson, Seung-Yong Choi, Mrinmoy Saha, Benjamin Wertz, Priyanka Trivedi, Chandrashekhar Korgaonkar, Harvey Chin, Onson Luong, Christos Kyratsous, William Olson

**Affiliations:** 1 Regeneron Pharmaceuticals, Inc., 777 Old Saw Mill River Road, Tarrytown, NY 10591 USA; 2 Abzena, 360 George Patterson Blvd, Bristol, PA 19007 USA

**Keywords:** Drug discovery and development, Drug development, Bacterial infection

## Abstract

For a number of years, antimicrobial resistance (AMR) has been a critical issue for humanity. Drug discovery efforts have been very limited and the spread of bacterial pathogens has over-run our traditional arsenal of antibiotics. Bacteria can involve to evade compounds that can halt their rapid growth. The authors have discovered a potent macrocycle derivative that when dosed concomitantly with the standard of care (SOC) antibiotic vancomycin, can clear methicillin resistant *Staphylococcus aureus* (MRSA) infections. In addition, we have probed the lead compounds in *Salmonella typhimurium* bacterial strains. In vitro, in vivo, and ADME data have been included to stress the virtues of this new antibiotic.

## Introduction

Antibiotic treatment failure due to antimicrobial resistance (AMR) has been a growing problem globally [https://www.who.int/news-room/fact-sheets/detail/antimicrobial-resistance]. The emergence of multidrug resistance of bacteria to known antibiotics further complicates the situation [1]. This health issue is being accentuated by the discoverers and manufacturers of antibiotics, pharmaceutical companies, having no incentive to discover and innovate. With limited antibiotic research being performed worldwide, we are at a critical juncture for human health.

Natural products have long been fertile grounds for discovering antibiotics. Since Fleming’s discovery of penicillin in 1929 [2] and Waksman’s soil actinomycetes discoveries of the 1940’s that ushered in systematic screening efforts by the pharmaceutical industry [3], natural products have dominated antimicrobial drug discovery efforts. At present, there’s one potent natural product that’s used as a drug of last resort against methicillin-resistant *Staphylococcus aureus* (MRSA) infections. Vancomycin, first isolated in 1953 from a Borneo soil sample [4] and marketed in 1958 by Eli Lilly, has other FDA-approved uses, namely, against *Staphylococcus enterocolitis*, pseudomembranous colitis, endocarditis, and *Clostridium difficile* [5]. In our research with vancomycin, we found that when dosed with a small amount of another derivative of a natural product macrocycle, the treatment can reduce infections in mice below the limits of detection, when compared to vancomycin alone.

## Results

### Screening, synthesis, in vitro potency, and structure-activity relationship

Screening a small and focused library of anti-infectives yielded a single series of very potent antibiotic macrocycles of the rifampicin class. After synthetic modifications, several analogs assayed inhibited growth of *S. aureus* both in a standard broth assay and an intracellular killing assay at sub-micromolar to nanomolar minimum inhibitory concentration (MIC) concentrations. We believe that the intracellular assay provides us with a measure of penetration, and thus, compounds to push forward into animals. The most potent and instructive compounds are listed in Table 1.

**Table 1:** Cellular activity of rifampicin-based antibiotics

		*S. aureus* NRS384		*Salmonella typhimurium* AR-0031		*Salmonella typhimurium* ATCC 14028	
No.	Structure	Broth MIC (M)	Intracellular MIC (M)	Broth MIC (M)	Intracellular MIC (M)	Broth MIC (M)	Intracellular MIC (M)
**1**		1.56E−08	1.00E−06	1.60E−05	No activity	1.60E−05	No activity
**2**		4.12E−07	1.00E−06				
**3**		4.57E−08	1.00E−06				
**4**		3.91E−09	4.00E−08	1.00E−06	3.00E−05	4.00E−06	1.00E−04
**5**		3.91E−09	4.00E−08	1.00E−06	3.00E−05	4.00E−06	>1e−4
**6**		6.25E−08	1.00E−06				

Synthesizing and assaying rifampicin analogs, we were able to remove the labile imine functionality and replace it with a stable benzo-oxazine ring system (rigidifying the molecule) and introduce the basic amine found in many potent antibiotics [6]. Although rifampicin **1** is potent in the broth assay, it lacks activity in the intracellular assay (Table 1). Our analogs (compounds **2,** **3,** **4**, and **5**) gain potency with the introduction of the fused 5 ring system and the alkylation of the basic amine in the broth assay. As the primary amine protons are replace with methyl groups, a log fold increase in potency is realized in the broth assay. However, the only compounds that gain potency in the intracellular killing assay are **4** and **5**, both tertiary amines, as expected.

Compound **6** shows the fine selectivity of the bacteria when a small change in the structure is introduced. By shifting the tertiary amine tail by one carbon position, it reduces the intracellular potency to rifampicin levels and causes a log reduction in the broth assay.

Taking rifampicin, compounds **4** and **5** into Gram-negative *Salmonella* strains AR-0031 and ATCC 14028, we see activity in both the broth and intracellular assays (Table 1). Although, not as potent as observed against *S. aureus*, they have an appreciable activity over and above rifampicin. Given these results, we believe that compounds **4** and **5** can have broader antibacterial properties.

### In vivo efficacy [7–10]

#### Median *S. aureus* NRS384 kidney burden in mice treated with rifampicin in combination with vancomycin

In this experiment, mice infected with *S. aureus* MSRA strain NRS384 were treated with vancomycin alone (control) or vancomycin in combination with 0.002 mg/kg to 25 mg/kg rifampicin.

**Fig. 1:**
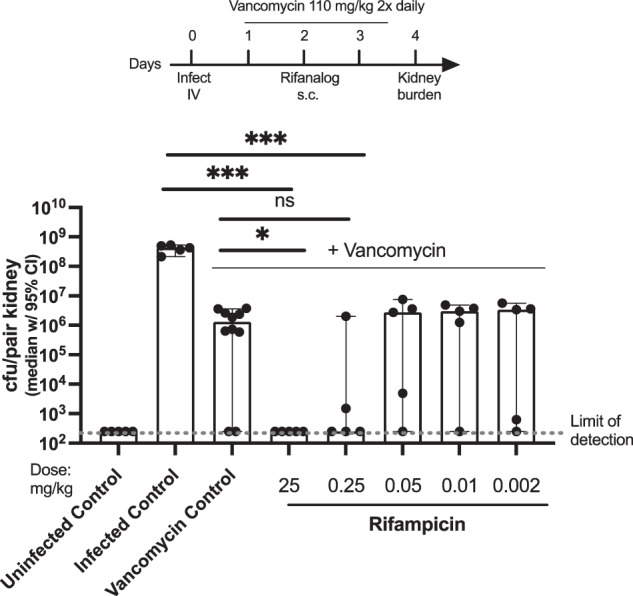
Mice infected with *S. aureus* MRSA strain NRS384 then treated with vancomycin (110 mg twice daily) and rifampicin. ***p < 0.001, *p < 0.05, ns = not significant (*t*-test). Figure adapted from PCT WO 2022204499

As shown in Fig. 1, intravenous (IV) infection with *S. aureus* MRSA strain NRS384 results in high bacterial burden in the kidneys. Vancomycin treatment alone results in a ~ 2–3 log reduction in *S. aureus* kidney burden. Combination treatment of rifampicin with vancomycin further reduces kidney burden. Rifampicin was effective in combination with vancomycin at 25 mg/kg and perhaps 0.25 mg/kg, but efficacy was reduced to vancomycin alone levels at lower doses.

#### Median *S*. aureus NRS384 kidney burden in mice treated with rifamycin analogs in combination with vancomycin

In this experiment, mice infected with *S. aureus* MSRA strain NRS384 were treated with vancomycin alone (control) or vancomycin in combination with 0.01–0.25 mg/kg of compounds **4** and **5**.

**Fig. 2:**
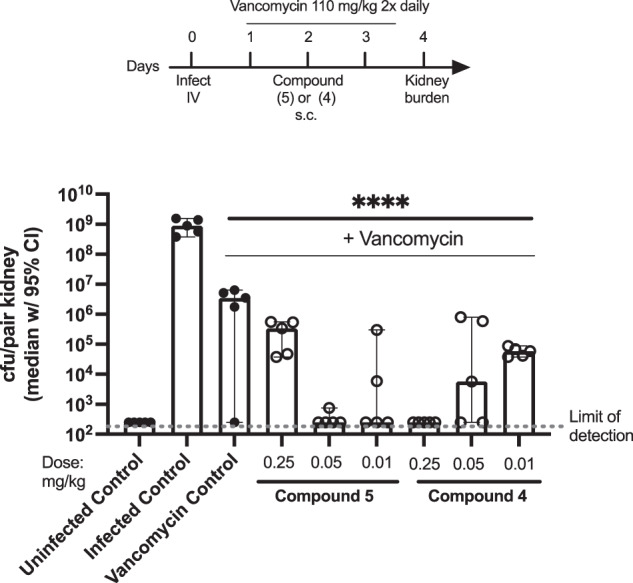
Mice infected with *S. aureus* MRSA strain NRS384 then treated with vancomycin (110 mg twice daily) and compounds **4** and **5**. *p* < 0.0001 (two-way ANOVA). Figure adapted from PCT WO 2022204499

As shown in Fig. 2, IV infection with *S. aureus* MRSA strain NRS384 results in high bacterial burden in the kidneys. Vancomycin treatment alone resulted in a ~ 2–3 log reduction in *S. aureus* kidney burden. Combination treatment of compounds **4** and **5** with vancomycin further reduced kidney burden.

Compounds **4** and **5** in combination with vancomycin are effective at reducing *S. aureus* kidney burden at lower doses than rifampicin in combination with vancomycin. For example, compound **5** significantly reduced *S. aureus* kidney burden to below the limit of detection even at the lowest tested dose of 0.01 mg/kg.

#### Median *S. aureus* NRS384 kidney burden in mice treated with rifampicin or compound 5 with or without combination with vancomycin

In this experiment, mice infected with *S. aureus* MSRA strain NRS384 were treated with 25 mg/kg rifampicin (control) or 0.002–0.25 mg/kg compound **5** in combination with or without vancomycin.

**Fig. 3:**
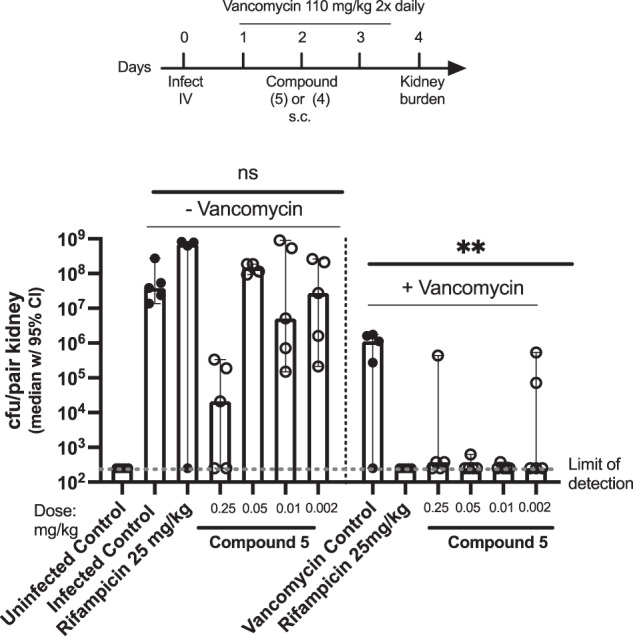
Mice infected with *S. aureus* MRSA strain NRS384 and treated with or without vancomycin (110 mg twice daily) and compound **5**. ns = not significant, **p < 0.005 (two-way ANOVA). Figure adapted from PCT WO 2022204499

As shown in Fig. 3, IV infection with *S. aureus* MRSA strain NRS384 results in high bacterial burden in the kidneys. Vancomycin treatment alone resulted in a ~ 2–3 log reduction in *S. aureus* kidney burden. Rifampicin reduced kidney burden at 25 mg/kg when combined with vancomycin but was ineffective as a monotherapy. Compound **5** significantly reduced *S. aureus* kidney burden by ~3 logs at 0.25 mg/kg when administered as a monotherapy.

As shown in Fig. 3, compound **5** is highly effective at low doses, including 0.05 and 0.002 mg/kg, at reducing the bacterial load to below the limit of detection when given in combination with vancomycin. Compound **5** in combination with vancomycin is more effective than either monotherapy, i.e., than compound **5** alone or vancomycin alone, in reducing the bacterial burden.

As shown in Fig. 3, the efficacy of compound **5** is enhanced by its use in combination with vancomycin. The efficacy of vancomycin is enhanced by its use in combination with compound **5**.

#### Median *S. aureus* N315 kidney burden in mice treated with rifampicin or compounds 4 and 5 in combination with vancomycin

Similar results were observed using another MRSA strain. In this experiment, mice infected with *S. aureus* MSRA strain N315 were treated with 25 mg/kg rifampicin (control), or 0.01–0.75 mg/kg compound **4**, or 0.002–0.25 mg/kg compound **5** in combination with vancomycin.

**Fig. 4:**
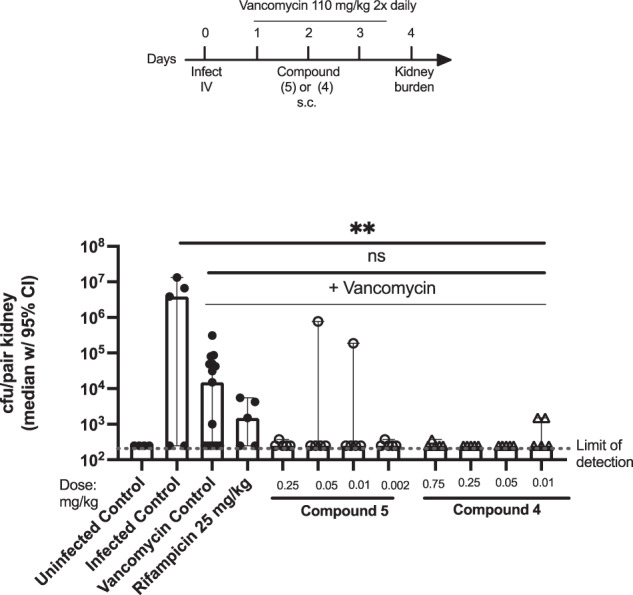
Mice infected with *S. aureus* MRSA strain NRS315 and treated with vancomycin (110 mg twice daily) and compounds **4** and **5**. ns = not significant,**p < 0.005 (two-way ANOVA). Figure adapted from PCT WO 2022204499

As shown in Fig. 4, IV infection with *S. aureus* MRSA strain N315 results in high bacterial burden in the kidneys. Vancomycin treatment alone resulted in a ~ 4 log reduction in *S. aureus* kidney burden. Combination treatment of 25 mg/kg rifampicin or 0.01–0.75 mg/kg compound **4** or 0.002–0.25 mg/kg compound **5** with vancomycin further reduced kidney burden to or near the limit of detection for the experiment.

Both leads, compounds **4** and **5**, were efficacious in combination with vancomycin to reduce the bacterial load to below the limit of detection at a dose of 0.01 mg/kg and 0.002 mg/kg, respectively, although the scatter in the vancomycin control group limited the statistical analysis.

### ADME

Compounds **4** and **5** were evaluated in standard assays for metabolic stability in rodent S9 microsomes, human plasma protein binding, permeability, and transporter activity, along with human CYP inhibition and induction (Tables 2–4). As shown in Table 2, plasma protein binding is in the range of 80-98% with compound **4** having the least binding. These values compare well to the approved antibiotics Rifampicin or Rifabutin. Table 2 also shows the rodent S9 microsomal stability for the compounds. Compounds **4** and **5** have half-lives greater than 120 min, which compares well to the approved antibiotics. Both compounds **4** and **5** exhibit no in vitro clearance in rat and mouse over 2 h which corresponds to low hepatic extraction. In Table 3 the permeability and transporter activity of compounds **4** and **5** are listed. Both leads have very good CACO-2 permeability ratios that rests between the values determined for rifabutin and rifampicin. As for the MDCK-MDR1 assay, again efflux ratios reside between the values determined for rifabutin and rifampicin. These values indicate that the compounds are effluxed by transporters. Finally, Table 4 shows the results for the CYP inhibition and induction activity for compounds **4** and **5**. Both leads inhibit all CYP isozymes tested, except for compound **4**, at uM values. Inhibition of CYP isozymes can potentially cause drug-to-drug interactions if the antibiotic is dosed high enough. This contrasts with both rifampicin and rifabutin that mainly are devoid of that inhibition. Table 4 also contains CYP induction activity for compounds **4** and **5**. Neither lead compound induces the CYP isozymes tested. However, rifabutin and rifampicin are strong inducers of these 3 CYP isozymes. The hERG potassium current was estimated to be >3 uM. The positive control, Cisapride, inhibited hERG current at 90 nM by 87.6%. It would not be expected that either lead would cause long QT syndrome.

**Table 2:** Human plasma protein binding and rodent S9 microsomal stability for compounds 4 and 5

Plasma Protein Binding				
Compound Number	% Protein Free	% Protein Bound	% Matrix Stability (6 h)	% Recovery (vs. T0 stability)
**4**	11.8 ± 2	88.2 ± 2	96.5	97.2
**5**	4.5 ± 0.5	95.5 ± 0.5	89.9	92.4
Rifabutin	10.4 ± 0.5	89.6 ± 0.5	104.9	98.2
Rifampicin	20.9 ± 0.6	79.1 ± 0.6	74.3	81.9

S9 Microsome Stability					
Compound Number	Species	T_1/2_ (min)	% Remaining at T_120_	CL_int_ (mL/min/kg)	CL_int_ (L/hr/kg)
**4**	CD-1 Mouse	>120	103.1	<45.5	<2.73
**5**	CD-1 Mouse	>120	106.4	<45.5	<2.73
Rifabutin	CD-1 Mouse	25	4.6	219	13.1
Rifampicin	CD-1 Mouse	>120	125.1	<45.5	<2.73
**4**	SD Rat	>120	112.4	<23.4	<1.4
**5**	SD Rat	>120	102.9	<23.4	<1.4
Rifabutin	SD Rat	1195	89	2.35	0.141
Rifampicin	SD Rat	>120	129.4	<23.4	<1.4

**Table 3:** Permeability and transporter activity for compounds 4 and 5

Compound Number	Mean P_app_ A-B (10^−6^ cm/s)	Mean P_app_ B-A (10^−6^ cm/s)	Mean (B-A/A-B) Efflux Ratio	Mean A-B % Recovery	Mean B-A % Recovery
**Caco-2: endogenous transporters**					
**4**	2.59	10.3	3.97	52.70%	66.30%
**5**	1.17	2.92	2.49	16.30%	58.30%
Rifabutin	11	7.15	0.648	64.80%	59.10%
Rifampicin	0.589	5.93	10.1	79.50%	80.80%
**MDCK-MDR1: overexpression of Pgp**					
**4**	1.38	18.9	13.7	89.10%	54.80%
**5**	1.33	10.4	7.8	48.20%	46.30%
Rifabutin	3.57	16.8	4.7	91.30%	67.90%
Rifampicin	0.287	6.25	21.8	76.50%	74.40%

**Table 4:** CYP inhibition and induction activity for compounds 4 and 5

CYP inhibition					
CYP Isozyme	CYP Substrate	IC50 (μM)			
		Cmpd 4	Cmpd 5	Rifabutin	Rifampicin
1A2	Phenacetin	10.9	2.02	ND	ND
2B6	Bupropion	11.6	2.98	ND	ND
2C8	Amodiaquine	6.76	1.99	ND	ND
2C9	Diclofenac	ND	4.65	ND	ND
2C19	Mephenytoin	9.99	2.54	ND	ND
2D6	Dextromethorphan	11.7	2.81	ND	ND
3A4	Midazolam	8.05	2.71	17.5	ND
3A4	Testosterone	5.66	2.15	36	ND

CYP induction				
Compound Number	Fold Induction (Enzyme Activity)			Average % Stability
	CYP 1A2	CYP 2B6	CYP 3A4	
**4**	0	0.0131	0.128	75.8
**5**	0	0.0131	0.128	53.7
Rifabutin	2	2.65	2.36	77.8
Rifampicin	1.33	10.8	11.8	85.2

*ND* Not Detected

### Toxicity

To investigate the toxicity of these antibiotic leads, a 2 week IV once-a-day (QD) dosing study was performed in Sprague Dawley rats to compare compounds **4** and **5** (see Table 5 for the study design). For compound **4**, approximately dose-proportional with the trend of less than dose proportional increase in C_max_ and AUC_tau_ was observed at low dose. The AUC or C_max_ of compound **4** was not impacted when coadministered with vancomycin. For compound **5**, greater than dose proportional increase in C_max_ between 0.1 and 10 mg/kg and AUC_tau_ between 1 and 10 mg/kg groups was observed. The AUC or C_max_ of compound **5** was not impacted when coadministered with vancomycin, except for Day 1 when a slightly greater C_max_ and AUC were observed on Day 1 in the animals coadministered compound **5** and vancomycin compared to the animals administered compound **5** only. No apparent accumulation was observed following 14 daily doses. No sex difference in exposure was observed. No apparent accumulation was observed following 14 daily doses. There were no body weight, body weight gains, or food consumption changes that were considered related to rifampicin, vancomycin, compounds **4** or **5** given alone, or in combination with vancomycin.

**Table 5:** Rat toxicity study design for once-a-day (QD) dosing of compounds 4 and 5

Group Number	Treatment	Dose Level (mg/kg/day)	Dose volume (mL/kg)	Dose concentration (mg/mL)	Tox Group (Main/Recovery)	
					Males	Females
1	Vehicle 1 (Compound **4**)	0	5	0	5/3	--
2	Vehicle 2 (Compound **5**)	0	5	0	5/3	--
3	Rifampicin	25	5	5	5/3	--
4	Vancomycin	110	5	22	5/3	--
5	Compound **4**	0.1	5	0.02	5/3	--
6	Compound **4**	1	5	0.2	5/3	5/3
7	Compound **4**	10	5	2	5/3	--
8	Compound **4** + vancomycin	1 + 110	2.5; 2.5	0.4; 44	5/3	--
9	Compound **5**	0.1	5	0.02	5/3	--
10	Compound **5**	1	5	0.2	5/3	5/3
11	Compound **5**	10	5	2	5/3	--
12	Compound **5** + vancomycin	1 + 110	2.5; 2.5	0.4; 44	5/3	--

Vehicle 1: 5% dextrose/citrate buffer (2:1), 0.7% Tween 80, pH 5.4

Vehicle 2: Captisol 30% (v/v) in Citrate buffer, pH 5 containing 0.5% (v/v) Tween 80

At the end of the treatment period, hematology changes were limited to minimal increases in reticulocyte counts (compound **4** at ≥0.1 mg/kg/day and compound **5** at 10 mg/kg/day). Changes observed in males given the combination of compounds **4** or **5** with vancomycin consisted of minimal decreases in red cell mass parameters (red blood cells, hemoglobin, and hematocrit), mild decreases in neutrophils counts and minimal increase in platelet counts. These changes were also noted in males given vancomycin alone and were therefore considered vancomycin-related. Compound **4** related changes in coagulation parameters was limited to minimal increases in fibrinogen in males given the combination, and were also noted in males given vancomycin alone, as such, the changes were attributed to vancomycin. Clinical chemistry changes consisted of minimal increases in phosphorus (compound **4** at ≥0.1 mg/kg/day), minimal decrease in albumin concentration (compound **4** at 10 mg/kg/day and the combination of compounds **4** or **5** with vancomycin), minimal increase in glucose concentration (compound **4** at 1 mg/kg/day and the combination of compound **4** with vancomycin) and minimal increase in urea nitrogen (combination of compound **4** with vancomycin). For compound **4**, at the end of the recovery period, additional changes observed consisted of minimal to mild decreases in globulin in males at ≥0.1 mg/kg/day and in males given the combination. In addition, there was an apparent minimal increase in alkaline phosphatase in males at ≥1 mg/kg/day and increase in glucose at ≥0.1 mg/kg/day. Additional changes observed in males given the combination included a minimal decrease in calcium after the 14-day recovery. Changes in coagulation parameters were no longer observed following the 14-day recovery period. For compound **5**, at the end of the recovery period all of the hematology changes were no longer observed with the exception of persistent increase in reticulocytes and red blood cell distribution width in males given 10 mg/kg/day and there were apparent minimal increases in platelet counts in males at ≥1 mg/kg/day and in males given the combination. There was an apparent minimal prolongation of activated partial thromboplastin time in males previously given 10 mg/kg/day following the 14-day recovery period. Decrease in globulin/increase in albumin/globulin ratio in males given 1 mg/kg/day, were still noted at the end of 14-day recovery period. A minimal decrease in alanine aminotransferase activity and mild increase in triglyceride concentrations were observed in males given the combination following the 14-day recovery period.

None of the compound **4** or **5** related findings were considered adverse. Overall, compounds **4** and **5** were well-tolerated. The no-observed-adverse-effect level (NOAEL) was considered to be 10 mg/kg/day, the highest dose administered for compound **4** and **5**.

## Discussion

The use of more than one drug to treat disease has been used in oncology where cancer cells have been difficult to control (e.g., CHOP for lymphoma) [11] and infectious disease where cocktails of antivirals have rendered deadly infections manageable (e.g., HIV) [12]. The authors present here a cocktail of 2 drugs that can treat MRSA infections with just one low dose of a rifampicin-based analog concomitantly dosed with the SOC antibiotic, vancomycin. Vancomycin, targeting lipid II, and the rifampicin-based compounds **4** and **5**, presumably targeting the DNA-dependent RNA polymerase, could be a potent combination to fight MRSA infections. This combination of antibiotics has been reported before [13, 14], however, our novel rifampicin-based antibiotics can reduce the bacterial CFUs below detectable levels at lower dose levels preclinically. It is known that vancomycin has poor cellular penetration [15] whereas compounds **4** and **5** have potent intracellular activity much greater than rifampicin. This synergy could prove valuable for other DNA-dependent RNA polymerase inhibitors.

Our in vitro results translated to our in vivo efficacy very well and, in fact, low doses of compounds **4** and **5** were needed to control infections using two *S. aureus* MRSA strains. This potency is very much needed since this class of antibiotics have clinical liabilities, namely, CYP induction that limits its use because of drug-to-drug interactions (DDI). Here, we provide alternatives to rifampicin or rifabutin given the ADME, toxicity profile, and superior potency of compounds **4** and **5**. Although, compounds **4** and **5** inhibit CYP isozymes, that liability can be mitigated by the very low and single doses of these antibiotics needed for efficacy.

AMR is a serious problem and with this report we discovered very potent antibiotics. However, a significant number of resources are needed to develop small molecule drugs. Partnership in pharmaceutical development is common in the industry and would be welcome for these antibiotics.

## Materials and methods

### Synthesis of rifampicin analogs

The methods used to synthesize, purify, and characterize the rifampicin analogs are detailed in the supplemental information Schemes S1–S4.

### *S. aureus* and *S. typhimurium* broth killing assay

To test the potency of rifamycin analogs of the disclosure in vitro, a broth growth inhibition assay was developed. For the assay, *S. aureus* NRS384 (BEI Resources), *S. typhimurium* AR-0031 (CDC & FDA Antibiotic Resistance Isolate Bank, Atlanta (GA): CDC 2018) (and *S. typhimurium* ATCC 14028 (American Type Culture Collection) were grown in Tryptic Soy Broth (TSB, Teknova) overnight, then sub-cultured 1:50 in fresh TSB and grown for an additional 2 h. The cultures were then pelleted via centrifugation and washed twice in PBS (Gibco). The cultures were then diluted to 1 × 10^6^ cfu/ml in TSB and 100 µl of the suspension was added per well to a 2 ml dilution plate in triplicate (Greiner Bio one). A dilution series of the indicated antibiotic was added 1:1 for a final starting concentration of 1 × 10^−5 ^M, then a 1:10 dilution for 1 × 10^−6 ^M, followed with 1:4 dilutions to include 2.5 × 10^−7 ^M, 6.25 × 10^−8 ^M, 1.56 × 10^−8 ^M, 3.91 × 10^−9 ^M, 9.77 × 10^−10 ^M, 2.44 × 10^−10 ^M, 6.1 × 10^−11 ^M, 1.53 × 10^−11 ^M, and 3.81 × 10^−12 ^M for a total of 11 points repeated 3 times. The plates were sealed and incubated at 37 °C with shaking for 24 h, then 150 µl of each sample was added to 96 well microtiter plates and OD_600_ was read on a Spectramax i3 Minimax 300.

### *S. aureus* and *S. typhimurium* intracellular killing assay

THP-1 monocytic cell line (American Type Culture Collection) was grown in media (RPMI + 10% FBS + 1% Penicillin/Streptomycin), then seeded at a density of 1e5 cells/well in a 96 well plate and differentiated into macrophages for 3 days prior to infection using 200 nM PMA. Overnight culture of *S. aureus* or *S. typhimurium* was grown in RPMI or TSB, respectively, washed twice with PBS and resuspended at 1e7 cfu/ml in PBS. THP-1 were washed with warm media (RMPI without FBS) to remove the Penicillin/Streptomycin and then infected with the bacterial suspension at a multiplicity of infection of 10:1 (*S. aureus*:macrophages) or 5:1 (*S. typhimurium*:macrophages). Plates were centrifuged at 300 × *g* for 5 min to synchronize adhesion of the bacteria to the macrophages, then incubated at 37 °C for 2 h for *S. aureus* infection or 1 h for *S. typhimurium* infection. Free-floating bacteria were removed by washing twice with warm media and the remaining extracellular bacteria were killed by addition of media containing gentamicin (50 ug/ml). After 1 h, media was aspirated and the indicated compound was added to the *S. aureus*-infected macrophages in a dilution series starting at 1e−6 M, with 1:5 dilutions for 6 points (1.0 × 10^−6 ^M, 2.0 × 10^−7 ^M, 4.0 × 10^−8 ^M, 8.0 × 10^−9 ^M, 1.6 × 10^−9 ^M, and 3.2 × 10^−10 ^M) or to the to the *S. typhimurium*-infected macrophages in a dilution series starting at 1e−4 M, with 1:3 dilutions followed by 1:10 dilutions for 6 points (1.0 × 10^−4 ^M, 3.0 × 10^−5 ^M, 1.0 × 10^−5 ^M, 1.0 × 10^−6 ^M, 1.0 × 10^−7 ^M, and 1.0 × 10^−8 ^M). The compounds were added in media containing 50 µg/ml gentamicin to prevent extracellular growth of bacteria. After 2 h, plates were washed twice with warm RPMI without FBS, and 100 μl of THP-1 lysis buffer (0.1% Triton in PBS) was added to each well. Bacterial survival was enumerated by colony forming units (cfu) through serial dilution and plating onto tryptic soy agar (TSA, Teknova) plates.

### *S. aureus* intravenous (IV) advanced disseminated infection mouse model

To test the efficacy of compounds **4** and **5** in combination therapy with vancomycin in vivo, a 4-day IV disseminated infection model was utilized. *S. aureus* MSRA strain NRS384 or N315 (BEI Resources), where indicated, was grown overnight in TSB and sub-cultured to mid-logarithmic phase. Bacteria were then washed twice with PBS and resuspended in PBS at a concentration of 1.5 × 10^^^8 cfu/ml NRS384 or 6.0 × 10^^^8 cfu/ml N315. Six-week-old Balb/c mice were then infected intravenously through the tail vein with 100 µl of the bacterial suspension, for a final infectious dose of 1.5 × 10^^^7 cfu/mouse NRS384 or 6.0 × 10^^^7 cfu/ml N315. From 1 to 3 days post-infection, indicated mice were injected subcutaneously with 110 mg/kg vancomycin twice daily where indicated. Compounds **4** or **5** were administered subcutaneously at the indicated dose 2 days after infection. Mice were monitored for weight loss and body conditioning score throughout the infection. At 4 days post-infection, mice were euthanized, and the *S. aureus* kidney burden was quantified by tissue homogenization followed by enumeration of cfu through serial dilution in PBS and plating onto TSA plates. Data points represent the kidney burden from individual mice tested. Statistical analysis was conducted with GraphPad Prism 9, Version 9.4.1.

### ADME

All standard ADME studies were conducted by Charles River Laboratories.

### Toxicity

A 2-week toxicity study of Compounds **4** and **5** with a 2-week recovery period in Sprague Dawley rats was performed at the Charles River Laboratories, Sherbrooke, QC, Canada. Compound **4** or Compound **5** (0, 0.1, 1, or 10 mg/kg/day) alone or in combination with vancomycin (110 mg/kg/day; SOC) and positive controls rifampicin (25 mg/kg/day) or vancomycin alone (110 mg/kg/day) were administered to male Sprague Dawley rats once daily for 14 days (14 doses) by intravenous (IV) injection. Compound **4** or **5** (1 mg/kg/day) was administered to female Sprague-Dawley rats once daily for 14 days (14 doses) by intravenous (IV) injection in order to evaluate any potential sex-related difference in exposure. A subset from each dose group was maintained for 14 days after the last dose to evaluate delayed toxicity and/or recovery. All animals were observed twice daily for mortality and signs of pain or distress. Detailed clinical observations, body weight measurements and food consumption were conducted weekly; post dose observations (30 min post dose) were conducted daily or as required. Clinical pathology evaluations (hematology, coagulation parameters, and serum chemistry) were performed using samples collected on the day of scheduled sacrifice (main and recovery). At the end of the 14-day dosing period, and at the end of the 14-day recovery period, necropsies were performed on all animals, selected organs were weighed, and tissues were examined macroscopically and microscopically. Toxicokinetic parameters were evaluated on Day 1 and 14. The study design is shown in Table 5.

## Conclusions

Antimicrobial resistance (AMR) has reached a crisis in recent years. We have developed ultrapotent antibiotic macrocycles for treating MSRA infections. The new rifampicin-based antibiotics have a synergistic effect once combined with the standard of care, vancomycin, that can neutralize the infection with very low single doses.

### Supplementary information

Supplemental Information

## References

[1] Nikaido H (2009). Multidrug Resistance in Bacteria. Annu Rev Biochem.

[2] Landau R, Achilladelis B, Scriabine A. Pharmaceutical Innovation: Revolutionizing Human Health. Chemical Heritage Foundation; 1999. p. 162.

[3] Lewis K (2020). The Science of Antibiotic Discovery. Cell.

[4] Shnayerson M, Plotkin M. The Killers Within: The Deadly Rise of Drug-Resistant Bacteria. Back Bay Books; 2003. ISBN 978-0-316-73566-7.

[5] Patel S, Preuss CV. Fidelia Bernice “Vancomycin” National Library of Medicine (2021) https://www.ncbi.nlm.nih.gov/books/NBK459263/.

[6] Richter MF (2017). Predictive compound accumulation rules yield a broad-spectrum antibiotic. Nature.

[7] Miller LS (2020). Development of a vaccine against Staphylococcus aureus invasive infections: Evidence based on human immunity, genetics and bacterial evasion mechanisms. FEMS Microbiol Rev.

[8] Fowler VG (2013). Effect of an investigational vaccine for preventing Staphylococcus aureus infections after cardiothoracic surgery: a randomized trial. JAMA.

[9] Surewaard BGJ (2016). Identification and treatment of the Staphylococcus aureus reservoir in vivo. JAMA.

[10] See PCT WO 2022204499.

[11] Fisher RI (1993). Comparison of a Standard Regimen (CHOP) with Three Intensive Chemotherapy Regimens for Advanced Non-Hodgkin’s Lymphoma. NEJM.

[12] Gorman C (1996). Man of the Year: The Disease Detective. Time Mag.

[13] Yu Y, Huang HL, Ye XQ, Cai DT, Fang JT, Sun J, Liao XP, Liu YH (2020). Synergistic Potential of Antimicrobial Combinations Against Methicillin-Resistant Staphylococcus aureus. Front Microbiol.

[14] Damasco PV, Cavalcante FS, Chamon RC, Ferreira DC, Rioja SS, Potsch MV (2013). The first case report of non-nosocomial healthcare- associated infective endocarditis due to methicillin-resistant Staphylococcus aureus USA400 in Rio de Janeiro, Brazil. Infection.

[15] Lehar SM (2015). Novel antibody–antibiotic conjugate eliminates intracellular S. aureus. Nature.

