# Supplementary material for: Potent Rifampicin derivatives can clear MRSA infections at single low doses when concomitantly dosed with Vancomycin
Source: J Antibiot (Tokyo). 2023 Oct 23;77(1):57–65. doi: 10.1038/s41429-023-00663-6 (PMC10766536; doi:10.1038/s41429-023-00663-6)
Supplement: Supplementary file 1 — Supplemental Information [file 41429_2023_663_MOESM1_ESM.docx]

**Supporting Information for**

**Potent Rifampicin derivatives can clear MRSA infections at single low doses when concomitantly dosed with Vancomycin.**

^a^Thomas Nittoli, ^a^Anna Brotcke Zumsteg, ^a^Abira Bandyopadhyay, ^a^Stephanie Federici, ^a^Alida Coppi, ^a^Susan Jorgenson, ^b^Seung-Yong Choi, ^b^Mrinmoy Saha, ^b^Benjamin Wertz, ^a^Priyanka Trivedi, ^a^Chandrashekhar Korgaonkar, ^a^Harvey Chin, ^a^Onson Luong, ^a^Christos Kyratsous, ^a^William Olson.

^a^Regeneron Pharmaceuticals, Inc., 777 Old Saw Mill River Road, Tarrytown, NY 10591 USA

^b^Abzena, 360 George Patterson Blvd, Bristol, PA 19007 USA

**Email:** Thomas.nittoli@regeneron.com

**SI Materials and Methods**

**General Methods.** All chemicals and solvents were purchased either from Sigma Aldrich or Fisher Scientific and were used without further purification except as specified. The catalyst [tBuBrettPhos Pd(allyl)]OTf (“Pd-175”) was purchased from Johnson Matthey. Rifamycin S was purchased from Bosche Scientific. ^1^H-NMR spectra were recorded on Varian 300 MHz and Varian 500 MHz NMR instruments. The chemical shifts (δ) are reported in ppm with respect to the NMR solvent signals used for the analysis. Chromatographic purities were determined on an Agilent 1100, 1260 Infinity, or 1200 Series LC/MS systems using Zorbax Eclipse™ XDB-C8 columns (4.6 x 150 mm, Agilent P/N 963967-906) and the following analytical HPLC method: injection volume 5 or 10 μL; flow rate 1 mL/min; 5-95% acetonitrile in water over 4 min; Agilent diode array detector at λ = 220 nm; room temperature. Preparative MPLC separations were performed on Teledyne Isco CombiFlash Rf instruments using the indicated stationary and mobile phases. High resolution mass spectra (HRMS) were recorded using an Agilent 6230 ESI-TOF LC/MS system equipped with a dual AJS electrospray ionization source. Low resolution mass spectrometry was performed using electrospray ionization sources with either single quadrupole or ion trap mass detectors.

**Syntheses of Rifamycin Analogs 2-4.**

**Scheme S1**: Syntheses of compounds **2-4**.

**Compound SI-1**: To a stirring solution of rifamycin S (2.00 g, 2.87 mmol, 1.0 equiv) under argon in 80 mL of toluene at room temperature was added 2-amino-5-bromophenol (0.54 g, 2.9 mmol, 1.0 equiv). The solution was stirred for 2 days at room temperature. The mixture was then evaporated to dryness and the black residue was dissolved in 20 mL of ethanol. After the solid material had dissolved, manganese dioxide (300 mg, 3.45 mmol, 1.2 equiv) was added in one portion to the ethanol solution. The thick mixture was stirred under argon for 15 h at room temperature. After filtration of insoluble materials using a Celite pad, the filtrate was evaporated under reduced pressure. The black residue was purified on a 120 g HP silica gel Gold RediSep column (gradient elution: 5 - 95% EtOAc in hexanes). The pure fractions were evaporated and dried in vacuo giving the title compound **SI**-**1** as a dark reddish solid (1.6 g, 65%). ESI-MS: calc’d for C_43_H_47_BrN_2_O_12_, 862.23; found 863.1 and 865.1 [M+H]^+^ (Pos). ^1^H NMR (500 MHz; DMSO-d6): δ 9.49 (d, *J* = 6.0 Hz, 1H), 7.92 (ddd, *J* = 3.6, 2.9, 1.8 Hz, 1H), 7.86 - 7.85 (m, 1H), 7.75 - 7.74 (m, 1H), 6.06 - 6.05 (m, 1H), 5.84 (dt, *J* = 2.6, 1.4 Hz, 2H), 5.25 - 5.23 (m, 2H), 4.80 (dt, *J* = 2.5, 1.0 Hz, 1H), 4.23 (td, *J* = 2.4, 1.0 Hz, 1H), 3.49 (d, *J* = 1.1 Hz, 1H), 3.10 - 3.09 (m, 2H), 3.03 (s, 3H), 2.79 (s, 1H), 2.19 (s, 3H), 2.01 (s, 4H), 1.96 (s, 4H), 1.81 (d, *J* = 2.2 Hz, 1H), 1.68 (s, 3H), 1.60 (dq, *J* = 2.8, 0.9 Hz, 1H), 1.48 (t, *J* = 1.4 Hz, 1H), 0.90 (dt, *J* = 2.1, 1.1 Hz, 2H), 0.84 (d, *J* = 7.1 Hz, 4H), 0.69 (dd, *J* = 2.2, 1.2 Hz, 5H).

**Compound SI-2**: An oven-dried 8 mL vial equipped with a magnetic stir bar was charged with compound **SI-1** (40 mg, 0.0463 mmol, 1.00 eq.), Fmoc-glycinol (131 mg, 0.463 mmol, 10.0 equiv), tBuBrettPhos Pd G3 (16 mg, 0.40 eq.), and K_3_PO_4_ (20.0 mg, 0.0942 mmol, 2.0 equiv). The reaction tube was capped with a rubber septum, evacuated, and backfilled with argon three times. Addition of 1,4-dioxane (2.0 mL) and the reaction was heated at 60 °C in a heating block under argon for 15 h. At the end of the heating period, the reaction was cooled to room temperature and then filtered through a pad of Celite®. The filter cake was rinsed with MeOH. The filtrate liquid was concentrated under reduced pressure, and the resulting residue was purified on a 50 g C18 Aq Gold column (gradient elution: 5 – 100% MeCN in water, 0.05% acetic acid in both). The product fractions were combined, frozen on dry ice, and lyophilized to give the title compound **SI-2** as a dark reddish solid (19 mg, 38%). ESI-MS: calc’d for C_60_H_63_N_3_O_15_ 1065.4; found 1066.4 [M+H]^+^ (Pos).

**Compound 2**: Compound **SI-2** of the preceding step (26 mg, 0.024 mmol) was dissolved in DMF (1 mL) and treated with a solution of piperidine (3.1 mg, 0.2 mL, 0.037 mmol) in DMF. The reaction was stirred under argon at ambient temperature. After 2 h, the mixture was loaded directly onto a 50 g C18 Aq Gold column (gradient elution: 0 – 100% MeCN in water, 0.05% acetic acid in both, over 30 min). The product-containing fractions were combined, frozen on dry ice, and lyophilized overnight to afford **2** as dark blue solid (9 mg, 44%). ESI-MS: calc’d for C_45_H_53_N_3_O_13_, 843.4; found 844.4 [M+H]^+^ (Pos), 842.3 [M-H]^-^ (Neg). ^1^H NMR (500 MHz; CD_3_OD): δ 7.83 (d, *J* = 8.8 Hz, 1H), 6.91 - 7.03 (m, 2H), 6.55 (s, 1H), 6.43 (d, *J* = 11.2 Hz, 1H), 6.21 - 6.30 (m, 2H), 4.98 - 5.08 (m, 2H), 3.76 (br. s, 3H), 3.43 - 3.47 (m, 1H), 3.41 (d, *J* = 5.37 Hz, 2H), 3.12 (br. s, 1H), 2.97 - 3.04 (m, 4H), 2.39 (br. s, 1H), 2.19 - 2.32 (m, 4H), 2.09 - 2.14 (m, 4H), 1.95 - 2.07 (m, 4H), 1.78 (s, 4H), 1.67 (d, *J* = 6.84 Hz, 1H), 1.31 (br. s., 2H), 0.97 (br. s, 8H), 0.66 - 0.85 (m, 4H), 0.08 (d, *J* = 5.5 Hz, 3H), -0.26 (d, *J* = 6.5 Hz, 3H).

**Compound SI-3**: An 8 mL oven-dried vial equipped with a stir bar was charged with compound **SI-1** (80.0 mg, 0.0926 mmol, 1.00 eq.), Fmoc-sarcosinol (275.0 mg, 0.9262 mmol, 10.0 eq.), tBuBrettPhos Pd G3 (40 mg, 0.5 eq.), and K_3_PO_4_ (39 mg, 0.19 mmol, 2.0 eq.). The reaction tube was capped with a rubber septum, evacuated, and backfilled with argon three times. Addition of 1,4-dioxane (3.0 mL) via syringe and the reaction was heated at 60 °C in a heating block under argon for 15 h. The reaction was cooled to room temperature, then filtered through a pad of Celite® and rinsed with MeOH. The crude material was concentrated in vacuo and purified on a 50 g C18 Aq Gold column (gradient elution: 5 – 100% MeCN in water, 0.05% acetic acid in both). The product fractions were combined, frozen on dry ice, and lyophilized to give the title compound **SI-3** as a dark reddish solid (49 mg, 49%). ESI-MS: calc’d for C_61_H_65_N_3_O_15_, 1079.4; found 1080.5 [M+H]^+^ (Pos).

**Compound 3**: Compound **SI-3** (49 mg, 0.045 mmol) was dissolved in DMF (1 mL) and treated with a solution of piperidine (7.7 mg, 0.091 mmol) in DMF (0.45 mL). The reaction stirred under argon at ambient temperature. After 2 h, the mixture was loaded directly on a 50 g C18 Aq Gold column and purified by MPLC (gradient elution: 0 – 100% MeCN in water, 0.05% acetic acid in both, over 30 min). The product-containing fractions were combined, frozen on dry ice, and lyophilized overnight giving the title compound as a dark blue solid (18 mg, 46%). ESI-MS: calc’d for C_46_H_55_N_3_O_13_, 857.3; found 858.3 [M+H]^+^ (Pos). ^1^H NMR (500 MHz; CD_3_OD): δ 7.84 (d, *J* = 8.79 Hz, 1H), 7.11 - 7.20 (m, 1H), 6.88 - 6.96 (m, 1H), 6.64 ( s, 1H), 6.42 (d, *J* = 10.26 Hz, 1H), 6.17 - 6.28 (m, 2H), 4.93 - 5.06 (m, 2H), 3.86 (br. s, 1H), 3.66 - 3.84 (m, 8H), 3.18 - 3.31 (m, 7H), 3.10 (br. s, 2H), 2.94 - 3.05 (m, 6H), 2.37 (br. s, 1H), 2.25 (d, *J* = 4.88 Hz, 4H), 2.05 - 2.22 (m, 7H), 1.85 - 2.05 (m, 7H), 1.78 (s, 6H), 1.65 (br. s, 1H), 1.30 (br. s., 2H), 0.95 (br. s, 8H), 0.82 - 0.92 (m, 4H), 0.78 (br. s., 1H), 0.70 (br. s, 1H), 0.03 (d, *J* = 5.86 Hz, 3H), -0.28 (d, *J* = 5.86 Hz, 3H).

**Compound 4:** A procedure developed by Buchwald, et al. for the cross-coupling of aryl bromides with primary alcohols^1^ was employed to form the aryl ethers. To an oven dried 8 mL vial equipped with a stir bar was charged **1** (40.0 mg, 0.0463 mmol, 1.00 eq.), 2-(dimethylamino)ethan-1-ol (42 mg, 0.46 mmol, 10.0 eq.), tBuBrettPhos Pd G3 (11.8 mg, 30 mol%), and NaOt-Bu (5.0 mg, 0.051 mmol, 1.1 eq). The reaction tube was capped with a rubber septum, then evacuated and backfilled with argon three times. Addition of 1,4-dioxane (2.0 mL) via syringe and the vial was heated at 55°C ± 5 °C in an oil bath under argon for 15 h. The mixture was cooled to room temperature and filtered through a pad of Celite®. The filter cake was rinsed with ethyl acetate. The filtrate liquid was concentrated to a residue under vacuum, and the material was purified on a C18 Aq Gold column (15.5 g, gradient elution: 10 – 100% MeCN in water with 0.05% acetic acid in both). The product fractions were combined, frozen on dry ice, and lyophilized to afford the compound **4** as a dark reddish solid (12 mg, 30%). HRMS: calc’d for C_47_H_57_N_3_O_13_ 871.3891; found 872.3941 [M+H]^+^ (Pos) and 894.3749 [M+Na]^+^ (Pos). ^1^H NMR (300 MHz; DMSO-d6) δ 9.40 (s, 1H), 7.87 (d, *J* = 8.9 Hz, 1H), 7.23-7.16 (m, 2H), 6.83 (dt, *J* = 2.3, 1.1 Hz, 1H), 6.23 (d, *J* = 4.6 Hz, 1H), 6.06 (dd, *J* = 5.9, 1.1 Hz, 1H), 5.82 (dd, *J* = 2.3, 1.5 Hz, 2H), 5.24 (dt, *J* = 1.4, 0.7 Hz, 1H), 4.83-4.75 (m, 1H), 4.24 (d, *J* = 29.9 Hz, 3H), 3.80 (d, *J* = 1.3 Hz, 1H), 3.03 (t, *J* = 0.5 Hz, 3H), 2.88 (s, 1H), 2.78 (t, *J* = 0.9 Hz, 2H), 2.67 (s, 2H), 2.22 (d, *J* = 3.7 Hz, 4H), 2.15 (s, 2H), 2.02 (s, 2H), 1.96 (d, *J* = 1.2 Hz, 2H), 1.90 (s, 1H), 1.68 (s, 2H), 0.85 (d, *J* = 6.7 Hz, 3H), 0.69 (t, *J* = 1.2 Hz, 3H).

**Synthesis of Rifamycin Analog 5.**

**Scheme S2**: Syntheses of compound **SI-6**.

**Scheme S3**: Syntheses of compound **5**.

**Compound SI-5**: A 500 mL round bottom flask equipped with a pressure-equalizing addition funnel and a magnetic stirbar was charged with 2,6-dimethoxyaniline (**SI-4,** 4.50 g, 29.4 mmol, 1.00 equiv) and dichloromethane (175 mL). The resulting solution was cooled to 0 °C using an ice bath and stirred vigorously as a solution of bromine (5.7 g, 1.8 mL, 36 mmol, 1.2 equiv) dissolved in an additional 12.5 mL dichloromethane was added dropwise to the cold solution. After the addition had completed, the mixture was warmed to room temperature and stirring was continued for 16.5 h. At this stage, the flask was cooled back to 0 °C and the reaction was quenched via the addition of 1 N aqueous sodium hydroxide (40 mL). Additional DI water was added (20 mL), and the organic phase was separated off. The aqueous layer was further extracted with 2x25 mL dichloromethane, and the combined organic layer was dried over sodium sulfate. The mixture was filtered, and the solvent was removed under vacuum to afford a dark red solid. The crude product was purified via MPLC (220 g HP Sil, 0-20% EtOAc/hexanes), affording compound **SI-5** as a colorless solid (4.15 g, 61%). ESI-MS: calc’d for C_8_H_10_BrNO_2_, 230.99; found 232.2 and 234.2 [M+H]^+^ (Pos).

**Compound SI-6**: A 500 mL round bottom flask was charged with 4-bromo-2,6-dimethoxyaniline (**SI-5**, 4.15 g, 17.9 mmol, 1.00 equiv), dichloromethane (50 mL), and a magnetic stirbar. The mixture was cooled to 0 °C using an ice bath and magnetic stirring was engaged. Neat boron tribromide (25 g, 9.3 mL, 5.5 equiv) was added very slowly with constant stirring. Stirring was continued as the ice bath melted, and the mixture warmed to room temperature naturally. After 16 h the ice bath was recharged, and the flask was cooled back to 0 °C. Deionized water (50 mL) was added very slowly, with constant stirring and the flask open. Following the addition of water, the mixture was stirred for 1 h at 0 °C. Saturated aqueous sodium bicarbonate solution (200 mL) was then added slowly, followed by additional dichloromethane (100 mL). The aqueous phase was separated, then frozen using dry ice and lyophilized. A green solid was obtained, which was washed with dichloromethane (2 x 100 mL) followed by ethyl acetate (2 x 50 mL). The wash liquids were decanted off, combined, and filtered. The solvent was removed under vacuum to afford compound **SI-6** as a colorless solid (2.79 g, 76%). ESI-MS: calc’d for C_6_H_6_BrNO_2_ 202.96; found 204.03 and 206.02 [M+H]^+^ (Pos).

**Compound SI-7**: A round bottom flask was purged with argon and charged with 2-amino-5-bromobenzene-1,3-diol (**SI-6**, 1.00 g, 4.90 mmol, 1.00 equiv), toluene (125 mL), and tetrahydrofuran (125 mL). The mixture was stirred at room temperature as solid Rifamycin S (3.41 g, 4.90 mmol, 1.00 equiv) was added in a single portion. Stirring was continued for 3 days, at which time the solvent was removed under reduced pressure to afford a black residue. The material was redissolved in ethanol (70 mL) and then manganese dioxide (682 mg, 7.84 mmol, 1.59 equiv) was added. After stirring for 5 h, the mixture was filtered through celite. The filtrate was concentrated under vacuum to a black residue, and the material was subjected to MPLC (220 g HP Sil, 10-60% EtOAc/hexanes) to afford compound **SI-7** as a green solid. (969 mg, 22%). ESI-MS: calc’d for C_43_H_47_BrN_2_O_13_ 878.23; found 879.69 and 881.69 [M+H]^+^ (Pos). ^1^H NMR (500 MHz; DMSO-d6) δ 10.22 (br. s., 1H), 9.52 (br. s., 1H), 7.43 (br. s., 1H), 7.35 (br. s., 1H), 6.04 (br. s., 1H), 5.83 (br. s., 2H), 5.21 (d, J = 6.35 Hz, 2H), 4.89 (t, J = 10.50 Hz, 1H), 4.16 (br. s., 1H), 3.51 (br. s., 1H), 3.15 (br. s., 2H), 3.02 (br. s., 4H), 2.80 (t, J = 8.55 Hz, 1H), 2.21 (br. s., 3H), 2.08 (br. s., 1H), 1.96 (s, 4H), 1.99 (s, 4H), 1.78 (br. s., 1H), 1.71 (br. s., 3H), 1.60 (br. s., 1H), 1.47 (br. s., 1H), 0.84 (d, J = 6.84 Hz, 6H), 0.69 (br. s., 6H).

**Compound 5**: A round-bottom flask was charged with **SI-7** (554 mg, 0.630 mmol, 1.00 equiv), [tBuBrettPhos Pd(allyl)]OTf (“Pd-175”, 49 mg, 63 μmol, 10 mol%), K_3_PO_4_ (271 mg, 1.28 mmol, 2.03 equiv), 2-(dimethylamino)ethan-1-ol (1.12 g, 12.6 mmol, 1.27 mL, 20.0 equiv), 1,4-dioxane (7 mL), and then 4Å molecular sieves (approximately 20% by volume). A stream of argon was bubbled through the mixture for 5 minutes and magnetic stirring was engaged. The mixture was heated to 70 °C and stirred continuously for 48 h. At the end of the heating period, the mixture was diluted with methanol (200 mL) and filtered through celite. The filtrate was concentrated to a reddish residue. The crude material was purified by MPLC (HP C18 Aq 100 g, 10-65% CH_3_CN/H_2_O with 0.05% acetic acid in both), affording compound **7** as a red solid (147 mg, 26%). HRMS: calc’d for C_47_H_57_N_3_O_14_ 887.3841; found 910.3745 [M+Na]^+^ (Pos). ^1^H NMR (500 MHz; DMSO-d6) δ 10.12 (br. s., 1H), 9.39 (br. s., 1H), 6.75 (br. s., 1H), 6.70 (br. s., 1H), 6.03 (br. s., 1H), 5.77 (d, J = 15.14 Hz, 1H), 5.21 (br. s., 1H), 4.83 - 4.90 (m, 1H), 4.15 - 4.30 (m, 2H), 4.08 (br. s., 1H), 3.53 (br. s., 1H), 3.29 (s, 1H), 3.16 (br. s., 1H), 3.03 (br. s., 3H), 2.87 (br. s., 1H), 2.79 (br. s., 1H), 2.62 - 2.71 (m, 2H), 2.36 (s, 1H), 2.23 (s, 6H), 2.19 (br. s., 3H), 1.93 - 2.11 (m, 7H), 1.91 (s, 1H), 1.76 (br. s., 1H), 1.69 (br. s., 3H), 1.53 - 1.65 (m, 1H), 1.50 (br. s., 1H), 1.32 - 1.45 (m, 1H), 0.76 - 0.94 (m, 6H), 0.68 (br. s., 5H).

**Synthesis of Rifamycin Analog 6.**

**Scheme S4**: Synthesis of compound **6**.

**Compound SI-8**: Rifamycin S (2.0 g, 2.9 mmol) was dissolved in 80 mL of toluene at room temperature and treated with 2-amino-4-bromophenol (0.54 g, 2.9 mmol). The solution was stirred for 2 days at room temperature. The mixture was evaporated to dryness and the residue was dissolved in 20 mL of ethanol. After the solid material had dissolved, 300 mg of manganese oxide (MnO_2_) was added in one portion to the solution. The mixture was stirred under argon for 15 h at room temperature. The insoluble materials were removed by filtering the mixture through a pad of Celite. The filtrate was evaporated under reduced pressure to afford a black residue. The residue was purified by MPLC on a 120 g HP silica gel Gold RediSep column (gradient elution: 5 - 95% EtOAc in hexanes). Pure fractions were evaporated and dried in vacuo giving the title compound **SI-8** as a dark reddish solid (1.5 g, 60%). ESI-MS: calc’d for C_43_H_47_BrN_2_O_12_, 862.23; found 863.1 and 865.1 [M+H]^+^ (Pos), 885.1 and 888.0 [M+Na]^+^ (Pos). ^1^H-NMR (500 MHz; CDCl_3_): δ 8.19-8.19 (m, 1H), 7.66-7.64 (m, 1H), 7.48 (s, 2H), 7.06 (s, 1H), 6.23-6.18 (m, 1H), 6.01 (d, *J* = 12.3 Hz, 2H), 5.06-5.05 (m, 1H), 4.98 (dd, *J* = 12.2, 7.1 Hz, 2H), 3.11 (s, 3H), 3.03-3.00 (m, 2H), 2.33 (s, 6H), 2.13 (s, 3H), 2.07 (s, 6H), 1.83 (s, 6H), 1.70 (s, 2H), 1.54 (s, 1H), 0.97 (d, *J* = 6.6 Hz, 3H), 0.80 (d, *J* = 5.2 Hz, 6H), 0.58-0.57 (m, 4H).

**Compound 6**: An oven-dried 8 mL vial was charged with compound **SI-8** (60 mg, 0.069 mmol, 1.0 eq), 2-(dimethylamino)ethan-1-ol (62 mg, 0.69 mmol, 10 eq), tBuBrettPhos Pd G3 (30 mg, 0.04 mmol, 0.5 eq), and K_3_PO_4_ (30 mg, 0.141 mmol, 2.0 eq.). The reaction tube was capped with a rubber septum, then evacuated and backfilled with argon three times and 1,4-dioxane was added (1.5 mL). The reaction mixture was heated at 60 °C in an oil bath under argon for 15 h. At the end of the heating period, the material was concentrated under vacuum and purified on a 50 g C18 Aq column (gradient elution: 10 - 95% MeCN in water, 0.05% acetic acid in both). The product fractions were combined, frozen on dry ice, and lyophilized. The dark red solid thus obtained was subjected to further purification by preparative HPLC using a Teledyne Isco EZ Prep instrument (Gemini C18 5µm, 150 mm x 30 mm, 10 - 95% MeCN in water, 0.05% AcOH) to afford compound **6** (4.5 mg, 7.4%). ESI-MS: calc’d for C_47_H_57_N_3_O_13_, 871.39; found 872.4 [M+H]^+^ (Pos). ^1^H NMR (500 MHz; DMSO-d6) δ 9.48-9.32 (m, 2H), 7.68-7.49 (m, 2H), 7.44-7.27 (m, 1H), 6.11-5.95 (m, 1H), 5.88-5.76 (m, 2H), 5.28-5.16 (m, 2H), 4.84-4.71 (m, 1H), 4.21-4.18 (m, 1H), 3.57-3.43 (m, 2H), 3.09-3.01 (m, 1H), 2.82-2.75 (m, 1H), 2.67 (dd, *J* = 15.5, 10.1 Hz, 3H), 2.29-2.23 (m, 13H), 2.19 (d, *J* = 0.6 Hz, 9H), 1.99-1.91 (m, 1H), 1.69 (s, 1H), 1.64-1.56 (m, 1H), 1.55-1.43 (m, 1H), 1.24 (s, 1H), 0.85-0.84 (m, 7H), 0.69-0.68 (m, 4H).

**References**

1. Zhang, H.; Ruiz-Castillo, P.; Buchwald, S. L. *Org. Lett.* **2018**, *20*, 1580.
